# A shore-based preliminary survey of marine ribbon worms (Nemertea) from the Caribbean coast of Colombia

**DOI:** 10.3897/zookeys.439.5965

**Published:** 2014-09-10

**Authors:** Jaime Gonzalez-Cueto, Sigmer Quiroga, Jon Norenburg

**Affiliations:** 1 Programa de Biología, Facultad de Ciencias Básicas, Universidad del Magdalena. Carrera 32 No. 22-08, Santa Marta, Colombia; 2 Smithsonian Institution, Department of Invertebrate Zoology, National Museum of Natural History, 10th and Constitution Ave, NW Washington, DC 20560-0163

**Keywords:** Nemertini, Rhynchocoela, Caribbean biodiversity, benthic species

## Abstract

A checklist of benthic ribbon worm species from the Caribbean coast of Colombia is presented, including synonyms, distributions, a photographic record, and the main morphologic characters of each species for a rapid identification. This is the first research focused broadly on nemerteans in Colombia. 54 specimens of nemerteans were hand-collected from the rocky littoral of two different localities, and identified according to personal experience and specialist literature. 13 species were found; of which 11 represent new records for the country. These species belong to eight different traditionally used families: Tubulanidae, Valenciniidae, Lineidae, Amphiporidae, Cratenemertidae, Emplectonematidae, Drepanophoridae and Ototyphlonemertidae. The most common and abundant species was *Dushia atra*. The biodiversity of nemerteans in Colombia seems to overlap with the nemertean fauna from Florida and Brazil, explained by the convergence of the North Brazil Current, Guiana Current, Caribbean Currents and the Panama-Colombia Contracurrent in the sampled region. The results of this work suggest that the Caribbean coast of Colombia is a region with a high diversity of nemerteans, and provide important taxonomic data for environmental assessments and future biological research.

## Introduction

Nemerteans, phylum Nemertea, also known as Nemertini, Rhynchocoela, or ribbon worms, comprise a group of bilateral, coelomate and non-segmented worms (Turbeville 2002; Thollesson and Norenburg 2003). The main synapomorphy supporting monophyly of the phylum is the presence of an eversible proboscis housed in a fluid-filled cavity, the rhynchocoel, which is considered to be a true coelom anatomically (*ibid.*). The proboscis, though structurally independent of the digestive system, is the primary means of prey capture by nemerteans. About 1275 species of nemerteans are considered validly named, most of which are found in marine environments (Kajihara et al. 2008). Marine nemerteans occur worldwide and inhabit almost all marine ecosystems from shallow water to the deep sea. Benthic species typically are slender and may be somewhat dorsoventrally flattened, and have the ability to stretch and contract their bodies extensively. They often are cryptic in habit and not frequently observed by non-specialists. Nevertheless, several species are known to have important effects as active predators, especially on mollusks, crustaceans and annelids (Roe 1976; Thiel and Kruse 2001; Caplins and Turbeville 2011). Nemerteans also have caught the attention of different biological fields such as regeneration (Coe 1934, 1943), developmental biology (Martindale and Henry 1995; Maslakova et al. 2004), genetics (Andrade et al. 2012; Chen et al. 2012), and pharmacology (Kem et al. 2006).

About 36 species have been recorded for the Caribbean Sea (Corrêa 1961, 1963; Kirsteuer 1973, 1974, 1977; Schwartz and Norenburg 2005). Three were recorded for Colombian coasts: *Ototyphlonemertes erneba* and *Ototyphlonemertes lactea* (Kirsteuer 1977) were reported from La Guajira, in the northeastern part of the country, whereas *Baseodiscus mexicanus* was recorded from the Pacific Coast (Coe 1940). However, in almost every study carried out on benthic ecosystems of Colombia, nemerteans have been mentioned to be an abundant component of the macrofauna communities (López 1981; Dueñas 1998; Vides 1999; Trujillo et al. 2009). They are recorded only as nemerteans in these studies, with no further taxonomic evaluation, due to the use of collection and fixation methods unsuitable for ribbon worms. Accurate identification of nemerteans is best done in the context of a regional synoptic survey based as much as possible on living specimens (Norenburg 2009). Color, eye-pattern, proboscis armature and, among small specimens, even some internal anatomical features are most reliably available from living specimens. Here, we start that process for Colombian nemerteans.

Detailed taxonomic understanding of animals also may require them to be properly fixed for histological examination and, increasingly, for genetic studies. There are few species-level identification keys to nemerteans, and those that do exist cannot be applied reliably beyond their region of origin. This reflects the paucity of experts available to make regional keys and also the low level of explicit morphological variation available in nemerteans, which results in extensive superficial similarity among species. Taxonomy, and therefore phylogeny, within the phylum also is poorly resolved, because many (perhaps most) nemertean species descriptions are inadequate and diagnoses for genera and families often are conflicting or insufficiently diagnostic (Gibson 1985; Schwartz and Norenburg 2001; Maslakova and Norenburg 2008).

A recent higher-level phylogeny of the nemerteans was proposed on the basis of DNA sequence data (Thollesson and Norenburg 2003) and resolved some deep and long-standing questions but some high-level relationships remain cloudy, whereas others are hostage to the analytical paradigms used (Andrade et al. 2012). The longest-standing traditional taxonomy divides the phylum into the two classes Enopla and Anopla. The class Enopla is characterized by the synapomorphy of central proboscis armature, as well placement of the mouth, which opens anteriorly either subterminally or joined with the rhynchodeum. In Anopla the mouth characteristically is ventral and post-cerebral and the proboscis lacks discrete central armature. Neither class is supported as monophyletic by recent molecular phylogenies. Within these classes, four orders – Hoplonemertea, Heteronemertea, Palaeonemertea and Bdellonemertea – were recognized for most of the last 100 years, based on disposition of body-wall musculature, nerve cords, blood vessels, and nature of proboscis armature (Hyman 1951; Gibson 1972). There is strong molecular evidence supporting monophyly of Hoplonemertea and Heteronemertea respectively, whereas Palaeonemertea consistently is found to be non-monophyletic, with at least some lineages basal within the phylum (Thollesson and Norenburg 2003; Andrade et al. 2012). Bdellonemertea has had a controversial history but now consistently falls within Hoplonemertea Monostilifera Distromatorhynchocoela (Thollesson and Norenburg 2003; Andrade et al. 2012). Thollesson and Norenburg (2003) proposed a new phylogeny of Nemertea, dividing the phylum into two clades Palaeonemertea (but with unknown exact membership) and Neonemertea, with the latter comprising the Hoplonemertea and the new clade Pilidiophora (= Heteronemertea + Hubrechtellidae) based respectively on absence or presence of a pilidium larva. This proposal was only partially supported by a more recent study (Andrade et al. 2012). For taxonomic purposes it remains most practical to refer to three main groupings: Palaeonemertea (though not monophyletic), Pilidiophora, and Hoplonemertea (Santos and Norenburg 2011).

Recent phylogenetic studies also re-enforce views by a number of nemertean systematists that several of the most species-rich genera (especially *Cerebratulus*, *Lineus*, *Micrura*, *Amphiporus*, and *Tetrastemma*) are non-monophyletic (Schwartz and Norenburg 2001; Strand and Sundberg 2005; Puerta et al. 2010; Andrade et al. 2012). That, in turn, results in a number of common families not being monophyletic, whereas monophyly of other families generally has not been tested. Some family designations are used here as accepted in recent literature or as best approximations in the case of taxa we could not identify to species. The latter can reflect either inability to match a worm to an existing description or the species may be undescribed. We use only family names for which there is phylogenetic evidence that they distinguish, for our purpose here, unique clades relative to each other.

Although in the last decades research in biodiversity of Colombia has increased, the phylum Nemertea remains one of its most neglected taxa (Díaz and Acero 2003). We present here the first study to target the phylum Nemertea in the country, though focused primarily on the region of Santa Marta, Colombia. We report here the occurrence of 12 named and six unidentified species of nemerteans, in addition to two named species previously recorded for the northeastern Caribbean coast of Colombia (Kirsteuer 1977).

## Collection sites and methods

**Figure 1.**
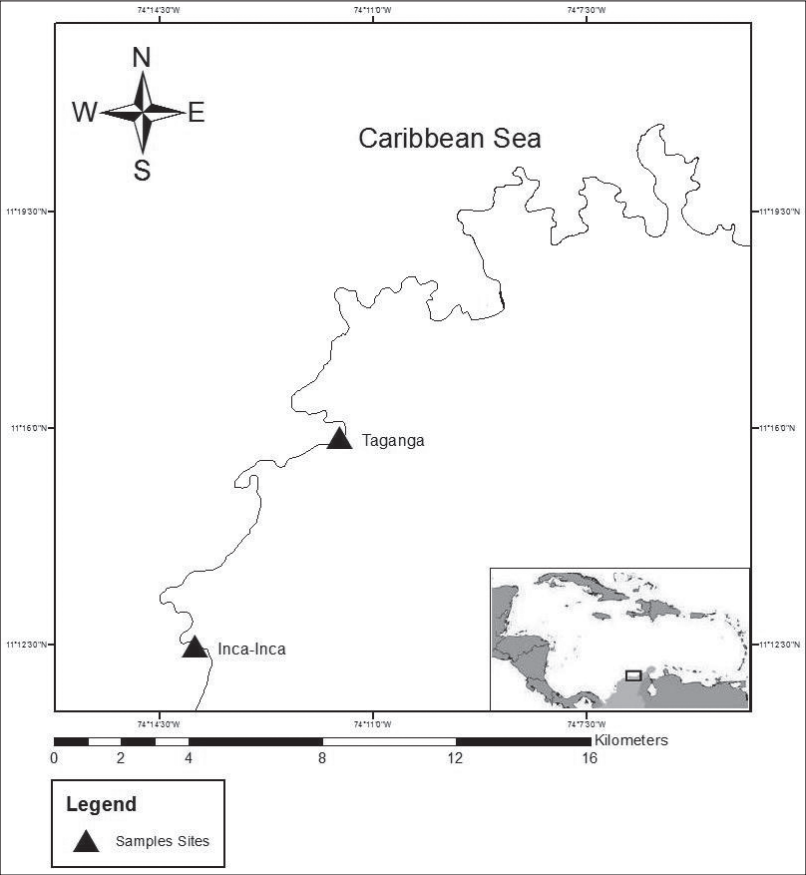
Map of the region of Santa Marta, Colombia, South America. The triangles show the sampled sites.

The material was collected from two different localities in the Santa Marta region on the Caribbean coast of Colombia: Inca-Inca and Taganga (Fig. 1).

The sites are in the bays formed by the extensions into the sea of the foothills of the Sierra Nevada de Santa Marta (SNSM), which is considered the highest coastal mountain range in the world. The common characteristic of these sites is the presence of a rocky littoral zone formed by metamorphic rocks interspersed with sandy beaches. There are two main climatic seasons – dry from December to April and rainy from May to November – influenced mainly by the Alisios winds (Dueñas 1998; Quiroga et al. 2004). During the dry season the region is affected by upwelling phenomena, producing changes in the temperature of the water and high biological productivity; in the rainy season there is a strong influence of fresh water from rivers draining the SNSM. The tidal range is 0.48 m (Garcia et al. 2011). Though the two sites are physically similar, they differ in anthropogenic impact. Inca Inca (74°14'W, 11°11'N) is located in one of the most important touristic centers in the Santa Marta region. Taganga (74°12'W, 14°15'N) is a traditional fishing bay where touristic activity has increased in the last decade and SCUBA activities have become economically important.

Specimens were collected from the rocky littoral zone. Three methods were used to look for the worms: inspecting the surface of rocks, breaking apart rocks when they had crevices, and leaving a portion of coarse substrate in a bowl of sea water so that worms were obligated to go up to the surface as the seawater de-oxygenated. Each specimen was relaxed in 7.5% magnesium chloride, measured and photographed *in vivo*, fixed in 10% buffered formalin and finally transferred to 70% ethanol after at least 24 hours of fixation. When necessary, histological sections were done at the level of the head and in the middle region of the body. For this, cross sections of paraplast embedded tissue were made and stained with hematoxylin and eosin. Taxonomic identification was based on Coe (1940, 1943, 1951), Corrêa (1954, 1961), Kirsteuer (1973, 1977), Riser (1991), Collin et al. (2005), Schwartz and Norenburg (2005), and Norenburg (2009). All the specimens were deposited at the CBUM.

## Results and discussion

A total of 54 specimens was collected, of which 46 were identified to 13 species, and 2 only to family; 4 individuals could not be identified and they are recorded as 4-eyed monostiliferans (Table 1). Two specimens self-destructed; one was a heteronemertean and the other a hoplonemertean (their tissues were preserved in absolute alcohol for future molecular study). All the named species found in this work have been recorded from other localities, including continental and insular Caribbean, Brazil, and Gulf of Mexico (Coe 1940, 1951; Corrêa 1954, 1961, 1963; Kirsteuer 1973, 1977; Collin et al. 2005; Schwartz and Norenburg 2005; Norenburg 2009), with the caveat that unknown levels of confidence accompany nemertean identifications based on morphology and often less than ideal original descriptions. Nevertheless, the species reported here seem to reflect minimal endemism and some species seem to have biogeographically complex distributions. The influence and combined effects of the North Brazil Current, Guiana Current, Caribbean Current and the Panama-Colombia Contracurrent could favor relatively wide dispersion of planktonic nemertean larvae and juveniles (Andrade et al. 2003; Okolodkov 2010). Potential hosts for *Carcinonemertes* and *Malacobdella*, both of which have worldwide distributions, were not sampled in this study. *Ototyphlonemertes erneba* and *Ototyphlonemertes lactea* were previously recorded in Colombia from the more northeastern Guajira region on the Caribbean coast (Kirsteuer 1977) but only *Ototyphlonemertes lactea* was found in the present survey, perhaps due to differences in habitats sampled and collecting methods.

**Table 1.** Nemerteans from Caribbean Coast of Colombia. The systematics is based on Thollesson and Norenburg (2003) and Chernyshev (2003). The specimens were deposited in the “Centro de Colecciones Biológicas de la Universidad del Magdalena”. Localities: (II) Inca-Inca, (TA) Taganga, (GU) Guajira. Habitats: (1) under rocks on muddy substrate, (2) under clean rocks, (3) interstitial, (4) rock crevices, (5) rocks with sponges.

Taxon	Locality	Habitat	Voucher	Synonyms
PALAEONEMERTEA: TUBULANIDAE				
*Tubulanus rhabdotus* Corrêa, 1954	II	1	CBUMAG:NEM00042	
HETERONEMERTEA: VALENCINIIDAE				
*Baseodiscus delineatus* (Delle Chiaje, 1825)	II, TA	2	CBUMAG:NEM:00002, CBUMAG:NEM:00008, CBUMAG:NEM:00012, CBUMAG:NEM00046, CBUMAG:NEM00051, CBUMAG:NEM00052	(a)
HETERONEMERTEA: LINEIDAE (*sensu lato*)				
*Dushia atra* (Girard, 1851)	II, TA	2, 3	CBUMAG:NEM:0003, CBUMAG:NEM:00006, CBUMAG:NEM00020, CBUMAG:NEM00027, CBUMAG:NEM00028, CBUMAG:NEM00029, CBUMAG:NEM00030, CBUMAG:NEM00031, CBUMAG:NEM00032, CBUMAG:NEM00033, CBUMAG:NEM00034, CBUMAG:NEM00035, CBUMAG:NEM00036, CBUMAG:NEM00037, CBUMAG:NEM00038	(b)
*Lineus stigmatus* Coe, 1951	TA	2	CBUMAG:NEM00050	
*Micrura ignea* Schwartz & Norenburg, 2005	II	1, 2	CBUMAG:NEM00001, CBUMAG:NEM00041	
HOPLONEMERTEA: MONOSTILIFERA				
*Amphiporus cruentatus* Verrill, 1879	II	2, 4	CBUMAG:NEM:00015, CBUMAG:NEM:00016	
*Amphiporus* cf. *ochraceus* (Verrill, 1873)	II	2, 4	CBUMAG:NEM:0011, CBUMAG:NEM00025, CBUMAG:NEM00026, CBUMAG:NEM00048	(c)
*Amphiporus texanus* Coe, 1951	II, TA	2, 4	CBUMAG:NEM00004 CBUMAG:NEM00017 CBUMAG:NEM00018 CBUMAG:NEM00019	
*Nemertopsis bivittata* (Delle Chiaje, 1841)	II	4	CBUMAG:NEM00048	(d)
*Ototyphlonemertes erneba* (Corrêa, 1950)	GA			
*Ototyphlonemertes lactea* (Corrêa, 1954)	TA, GA	3	CBUMAG:NEM00054, CBUMAG:NEM00055	(e)
*Zygonemertes fragariae* Corrêa, 1954	II	2	CBUMAG:NEM00040	
*Zygonemertes virescens* (Verrill, 1879)	II	2,4	CBUMAG:NEM:00007, CBUMAG:NEM:00009, CBUMAG:NEM:00014, CBUMAG:NEM:00021 CBUMAG:NEM:00022, CBUMAG:NEM:00023	(f)
4-eyed monostiliferan sp.1	TA	2	CBUMAG:NEM:00010, CBUMAG:NEM00024	
4-eyed monostiliferan sp.2	II	2	CBUMAG:NEM:0005	
4-eyed monostiliferan sp.3	TA	2	CBUMAG:NEM00044	
Cratenemertidae sp.	TA	2, 5	CBUMAG:NEM00043, CBUMAG:NEM00049	
HOPLONEMERTEA: POLYSTILIFERA: REPTANTIA				
*Punnettia* cf. *natans* (Kirsteuer, 1973)	TA	5	CBUMAG:NEM00045	(g)

The following taxonomic key (see below) and the descriptions of the main characteristics, together with photographs of the different species, provide a tool for rapid visual identification of the nemerteans found in this survey of the Santa Marta region of Colombia. That for *Ototyphlonemertes erneba* is based on published descriptions.

### Key to shoreline live Nemertea of Caribbean Colombia

**Table:** 

1a	Mouth ventral and posterior to brain; proboscis without modified middle region bearing armature	“Class” Anopla 2
1b	Mouth antero-terminal or subterminal and usually sharing a common opening with the rhynchopore but rarely the two open separately; proboscis with modified middle region bearing armature of one or more stylets	“Class” Enopla 6
2a	Cephalic lobe (head) demarcated posteriorly with pair lateral vertical furrows (may be indistinct)	3
2b	Cephalic lobe lateral margins each a distinct longitudinal furrow	Lineidae 4
3a	Ground color pale ochre; numerous irregularly spaced blackish-brown rings encircle body – most anterior interrupted by mouth, fourth bears pale, often indistinct, oval lateral sense organ	*Tubulanus rhabdotus*
3b	Ground color pale, milky tan; numerous reddish brown short but irregular length longitudinal lines cover dorsal surface and may extend ventrally; cephalic furrows with orthogonal secondary furrows	*Baseodiscus delineatus*
4a	Caudal cirrus present in adults but may be very small	5
4b	Caudal cirrus lacking in adults; blackish-brown ground color with numerous pairs of white markings dorsally along length of body; anterior 2/3 of cephalic lobe white with symmetrical brown patterning	*Lineus stigmatus*
5a	Body uniformly blackish except for white anterior margin of cephalic lobe and white caudal cirrus; mouth longer than relaxed width of worm	*Dushia atra*
5b	Body uniform bright orange anteriorly, grading to yellowish posteriorly	*Micrura ignea*
6a	Proboscis armature a fig with numerous tiny tack-like stylets; also numerous sacs of accessory stylets; long and wide oblique cephalic furrows with orthogonal secondary furrows; numerous conspicuous ocelli set in approximately 4 rows	*Punnettia* cf. *natans*
6b	Proboscis armature a distinct single stylet resting on a basis, usually with a pair of sacs containing accessory stylets	7
7a	Ocelli present (not always evident)	9
7b	Ocelli absent; statocyst present	*Ototyphlonemertes* 8
8a	Statocyst with usually 3 statolith granules; stylet smooth; papillae at anterior of anterior proboscis chamber each with rod-shaped inclusion	*Ototyphlonemertes erneba*
8b	Statocyst with about 12 statolith granules; proboscis extremely short (about length of head); stylet helically sculpted; cerebral organs and cephalic cirri absent	*Ototyphlonemertes lactea*
9a	Four ocelli set as rectangle	Unidentified monostiliferan spp 1–3 (see text)
9b	More than four ocelli (not always evident)	10
10a	Blood vessels conspicuously filled with red corpuscles	*Amphiporus cruentatus*
10b	Blood vessels “colorless”	11
11a	Body uniformly dark brown and opaque, cephalic lobe rimmed by white “halo”; about 6 ocelli along each antero-lateral margin visible only in specimens squeezed under coverslip	*Amphiporus texanus*
11b	Ocelli visible without squeezing specimen	12
12a	Ocelli extend laterally next to and posterior to cerebral ganglia; armature basis concave or flat posterior margin; epidermis contains small, intracellular, crescent-shaped hooks (requires squeezing specimen under coverslip and compound microscopy at 200–400×)	*Zygonemertes* 13
12b	Ocelli all precerebral; armature basis convex posterior margin	14
13a	Body pale to dark green as adult, may be grayish-white as juvenile	*Zygonemertes virescens*
13b	Body uniformly rosy pink	*Zygonemertes fragariae*
14a	Ocelli small, 6-10 in a pair of rows parallel to and near lateral margins of head; body uniformly translucent milky gray to yellowish, and cerebral ganglia with orange hue	*Amphiporus* cf. *ochraceus*
14b	Ocelli large, about 26 set in four groups but may appear to form one large group along each lateral margin of the head; body relatively opaque rosy red; armature basis very rounded and short relative to stylet; good swimmer when irritated	Cratenemertidae sp. 1

**Figure 2.**
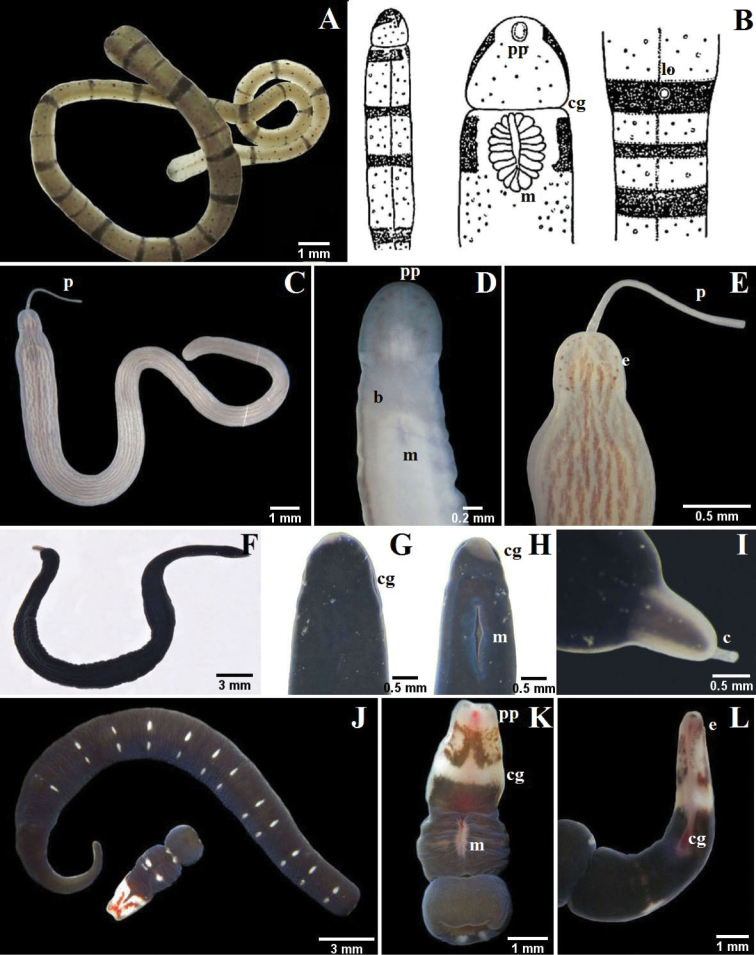
**A–B** *Tubulanus rhabdotus*: **B** detail of the head, mouth and lateral organ (modified from Corrêa 1954) **C–E** *Baseodiscus delineatus*: **C** entire specimen, the worm has expulsed the proboscis **D** ventral detail of the head **E** dorsal detail of the head **F–I** *Dushia atra*: **G** dorsal detail of the head **H** ventral detail of the head **I** detail of the tail showing the caudal cirrus **J–L** *Lineus stigmatus*: **J** entire specimen, the worm autotomized **K** ventral detail of the head **L** lateral detail of the head. *b* brain, *c* cirrus, *cg* cephalic grooves, *e* eyes, *lo* lateral organ, *m* mouth, *p* proboscis, *pp* proboscis pore.

**Figure 3.**
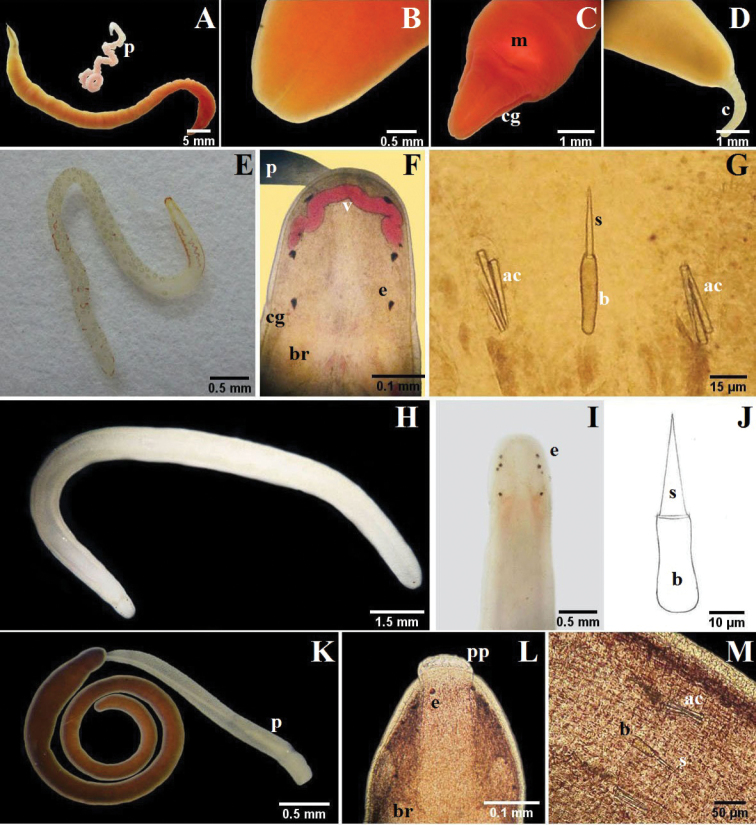
**A–D** *Micrura ignea*: **A** entire specimen, the worm has expulsed the proboscis **B** dorsal detail of the head **C** ventral detail of the head **D** detail of the tail **E–G** *Amphiporus cruentatus*: **F** dorsal detail of the head **G** detail of the stylets **H–J** *Amphiporus* cf. *ochraceus*: **I** dorsal detail of the head **J** drawing of the stylet **K–M** *Amphiporus texanus*: **K** entire worm **L** dorsal detail of the head **M** detail of the stylets. *ac* accessory stylet, *b* base of the stylet, *br* brain, *c* cirrus, *cg* cephalic grooves, *e* eyes, *m* mouth, *p* proboscis, *pp* proboscis pore, *s* sylet, *v* blood vessel.

**Figure 4.**
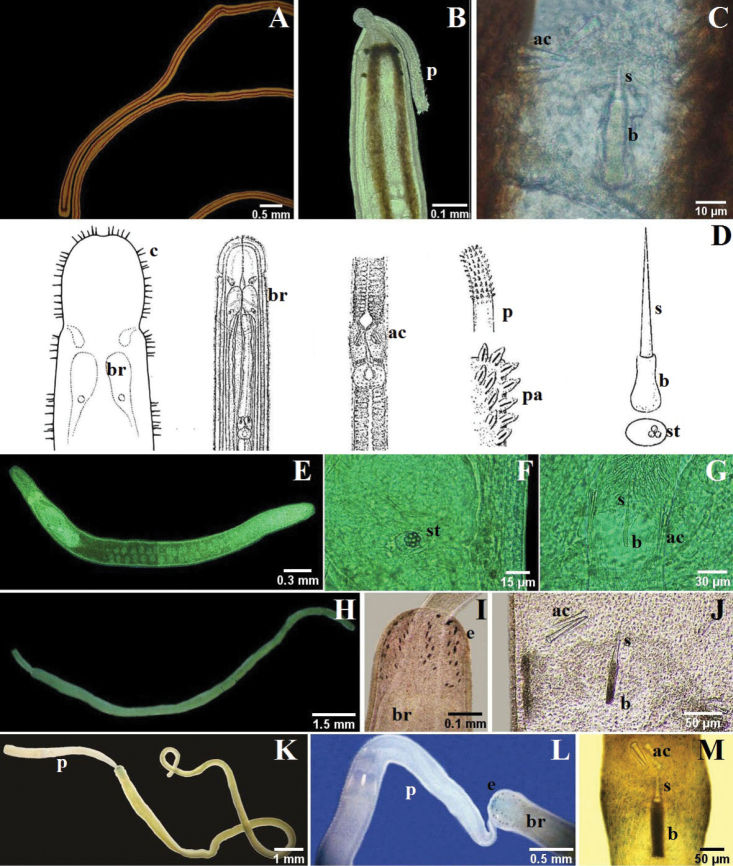
**A–C** *Nemertopsis bivittata*: **B** dorsal detail of the head and proboscis, **C** detail of the stylets **D** *Ototyphlonemertes erneba* (modified from Corrêa 1950 and Kirsteuer 1977) **E–G** *Ototyphlonemertes lactea*: **E** entire worm **F** detail of the statocysts **G** detail of the stylets **H–J** *Zygonemertes fragariae*: **H** entire worm **I** detail of the head **J** detail of the stylets **K–M** *Zygonemertes virescens*: **K** entire worm **L** detail of the head **M** detail of the stylets. *ac* accessory stylet, *b* base of the stylet, *br* brain, *c* sensorial cirrus, *e* eyes, *p* proboscis, *pa* proboscis papilla, *s* sylet, *st* statocysts.

**Figure 5.**
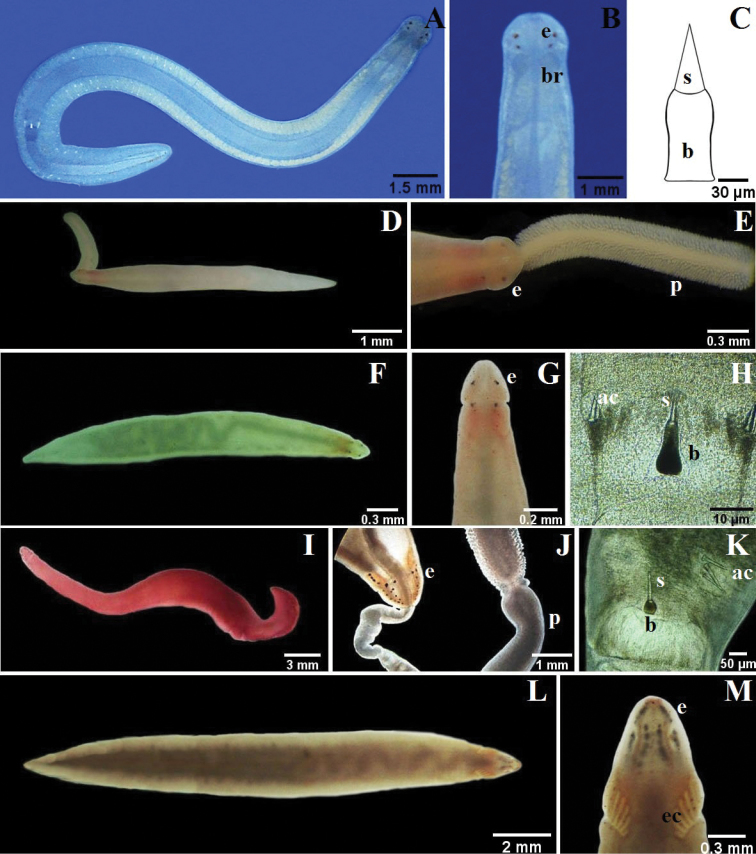
**A–C** 4-eyed monostiliferan sp. 1: **B** dorsal detail of the head **C** drawing of the stylet **D–E** 4-eyed monostiliferan sp. 2: **E** dorsal detail of the head and proboscis **F–H** 4-eyed monostiliferan sp. 3: **G** dorsal detail of the head **H** detail of the stylets **I–K** Cratenemertidae sp.: **J** dorsal detail of the head and proboscis **K** detail of the stylets **L–M** *Punnettia* cf. *natans*: **M** dorsal detail of the head. *ac* accessory stylet, *b* base of the stylet, *br* brain, *e* eyes, *ec* epithelial crests, *p* proboscis, *s* sylet.

The mouth is ventral and posterior to the cerebral ganglia in Palaeonemertea and Heteronemertea (e.g., Fig. 2D, K). In all the Hoplonemertea Monostilifera encountered here the mouth and proboscis share a common pore, the rhynchopore, located at or subterminal to the tip of the head (NB: they open separately in most polystiliferans and in a few monostiliferans, but both openings are at the tip of the head). In lineid Heteronemertea (generally and in all specimens encountered here) a more or less deep furrow extends longitudinally along each side of the head and often is referred to as a cephalic slit, groove or furrow; the cerebral organ pore opens at its posterior. Cephalic furrows (when present) of tubulanid palaeonemerteans, baseodiscid heteronemerteans and hoplonemerteans are shallow and vertical or oblique, at the sides of the head near the cerebral ganglia, and they may be inconspicuous; the cerebral organ pore opens into the middle of each furrow. These furrows might best be referred to as cerebral organ furrows to distinguish them from a circumferential cephalic groove; a shallow epidermal groove that encircles the body and demarcates the “head” from the foregut region and found in most benthic Hoplonemertea and a very few Palaeonemertea and Pilidiophora. It commonly is post-cerebral and in Hoplonemertea usually takes the form of a dorsal posteriorly directed “V” and a ventral anteriorly directed “V”. The hoplonemertean proboscis when everted reveals a characteristic cylindrically uniform coating of more or less conspicuous epidermal papillae, whereas the anoplan proboscis generally lacks conspicuous papillae and often is bilaterally differentiated. The mid-region of the monostiliferan hoplonemertean proboscis is conspicuously differentiated into a bulb-like structure posteriorly and an anterior diaphragm bearing a basis with stylet and two or more sacs containing accessory stylets (e.g., Fig. 3G, M, P; 4C, G, J, M; 5H, K), whereas the mid-proboscis of polystiliferan Hoplonemertea is inconspicuously differentiated and bears an ovoid basis with multiple tiny stylets that may be very difficult to observe even with the 40× objective of a compound microscope. Measurements given below are from animals collected in this study.

### Synonyms

(a) *Baseodiscus delineatus* (Delle Chiaje, 1825): *Baseodiscus curtus*, *Baseodiscus delineatus* var. *curta*, *Baseodiscus delineatus* var. *curtus*, *Baseodiscus insignis*, *Borlasia carmelina*, *Eupolia amboinensis*, *Eupolia ascophora*, *Eupolia curta*, *Nemertes delineatus*, *Nemertes delineatus*, *Polia delineata* Delle Chiaje, 1825 (Gibson 1995, 2014a).

(b) *Dushia atra* (Girard, 1851): *Cerebratulus ater*, *Lineus ater*, *Meckelia atra* Girard, 1851 (Norenburg 2009).

(c) *Amphiporus* cf. *ochraceus* (Verrill, 1873): *Cosmocephala ochracea* Verrill, 1873 (Gibson 1995).

(d) *Nemertopsis bivittata* (Delle Chiaje, 1841): *Eunemertes peronea*, *Nemerteopsis peronea*, *Nemertes peronea*, *Nemertopsis peronea*, *Omatoplea peronea*, *Ommatoplea peronea*, *Polia bivittata* Delle Chiaje, 1841; *Prosorhochmus bistriatus*, *Prosorochmus bistriatus* (Gibson 1995).

(e) *Ototyphlonemertes lactea* Corrêa, 1954: *Norenburgia lactea* Chernyshev, 1993 (Gibson 1995).

(f) *Zygonemertes virescens* (Verrill, 1879): *Amphiporus agilis*, *Amphiporus virescens* Verrill,1879; *Nemertes verrilli*, *Ophionemertes agilis* (Gibson 1995).

(g) *Curranemertes natans* Kirsteuer, 1973.

### Palaeonemertea: Tubulanidae

#### Tubulanus rhabdotus

Taxon classificationAnimaliaPalaeonemerteaTubulanidae

Corrêa, 1954

Fig. 2A, B

##### Description.

One specimen up to about 30 mm long and 1 mm wide; dorsoventrally flattened, cephalic lobe broadly rounded anteriorly; caudal terminus blunt. Ochre with numerous small dark dots arranged in longitudinal lines, dark brown rings of differing thickness spaced irregularly along body, first four rings thickest. Prominent but short, vertical cerebral organ furrows demarcate head from rest of body. Eyespots lacking. Mouth ventral, just posterior to cephalic grooves. Proboscis pore subterminal. Lateral sensory organ present in fourth ring. Worm secretes and lives in soft tube of honey color.

##### Distribution.

Curaçao (Corrêa 1963); Florida and Virgin Islands, USA (Corrêa 1961); São Paulo, Brazil (Corrêa 1954); Santa Marta, Colombia.

### Heteronemertea: Valenciniidae

#### Baseodiscus delineatus

Taxon classificationAnimaliaHeteronemerteaValenciniidae

(Delle Chiaje, 1825)

Fig. 2C–E

##### Description.

Six specimens up to about 50 mm long, 2 mm wide; dorsoventrally flattened; cephalic lobe broadly rounded. Milky ground color; dorsally with abundant short interrupted reddish to brown longitudinal lines, paler or completely absent on ventral surface. Shallow, cerebral organ furrows post-cerebral, vertical and slightly oblique, with inconspicuous, orthogonally oriented secondary furrows. Numerous ocelli arranged irregularly along antero-lateral margin of head. Mouth ventral, posterior to cerebral organ furrows. Proboscis pore at anterior of head; proboscis short and thin.

##### Distribution.

This species seems to be circumglobal in tropical and subtropical seas (Norenburg 2009).

### Heteronemertea: Lineidae (*sensu lato*)

#### Dushia atra

Taxon classificationAnimaliaHeteronemerteaLineidae

(Girard, 1851)

Fig. 2F–I

##### Description.

Fifteen specimens up to about 160 mm long, 2.5 mm wide; dorsoventrally flattened; head elongate, can be pointy; short, slender caudal cirrus present. Black body, lips of cephalic furrows, tip of head and tail grayish or milky white. Deep cephalic furrows form lateral margins of head. Ocelli lacking. Mouth ventral, a large longitudinal slit posterior to cephalic furrows. Proboscis long, yellow, with smooth surface when everted.

##### Distribution.

Curaçao (Corrêa 1963), Gulf of Mexico (Norenburg 2009); Santa Marta.

##### Comments.

This was the most frequently found species, though not necessarily the most abundant; we did not do quantitative sampling in this study. Given the wide regional distributions of other nemerteans in this and other recent studies (e.g., Corrêa 1961, 1963; Norenburg 2009) one expects such a common species to be identifiable as a regionally known species. Many of the early descriptions, however, are based on highly contracted and, often, fragmented specimens and lack observations from life (e.g., Verrill 1900; Coe 1901; Stiasny-Wijnhoff 1920). Presence or absence of a caudal cirrus is unreliably known for several named species from the region that are more or less blackish. The genera *Cerebratulus* and *Micrura*, both considered to have a caudal cirrus as a matter of diagnosis, are known among nemertean specialists to be fraught with taxonomic inconsistencies (see, e.g., Schwartz and Norenburg 2001, 2005). The presence or absence of a caudal cirrus seems, based on DNA data, to be an unreliable diagnostic for those genera (Schwartz 2009). Possible Caribbean options for our species include *Cerebratulus leucopsis* (Coe 1901), reported to have a caudal cirrus, and *Corsoua kristenseni* Corrêa, 1963, reported to lack a caudal cirrus. We believe that the former may be synonymous with *Dushia atra* *sensu* Corrêa (1963) from Curaçao. *Corsoua kristenseni* has a small mouth and occurs in mangrove habitat, whereas *Dushia atra* *sensu* Corrêa (1963) has a large mouth and occurs at the high-water line in clean sand under rubble, as do our specimens, though Corrêa (1963) does not mention a caudal cirrus. Our material consistently has a small caudal cirrus but it could easily be missed in preserved specimens. Pending anatomical studies, we assign our specimens to *Dushia atra* *sensu* Corrêa (1963). A potential taxonomic problem remains in that Girard (1893) describes his specimens as having been dredged from deep water off Cape Florida, which is at strong variance with the very narrowly constrained habitat of our specimens and those found on other Caribbean islands (JLN, pers obs).

#### Lineus stigmatus

Taxon classificationAnimaliaHeteronemerteaLineidae

Coe, 1951

Fig. 2J–L

##### Description.

One specimen up to about 30 mm long, 2 mm wide; dorsoventrally flattened, anterior margin somewhat squared, posterior end tapered, caudal cirrus absent. Body dark violet or dark olive green; paired, widely spaced, transversely elongate white markings dorsally, each pair part of barely perceptible thin white ring encircling body; anterior two-thirds of head white with prominent brown pigment patterning, including a V-shaped marking dorsally and ventrally, each pointed anteriorly. Deep cephalic furrows form lateral margins of head. About 20 to 30 small ocelli disposed in a single irregular line on each side of head along anterior third of cephalic furrow. Mouth ventral, a large longitudinal slit posterior to cephalic furrows. Cephalic ganglion visible through body wall as a pink mass.

##### Distribution.

Florida (Coe 1951), Belize (JLN, pers obs); Santa Marta, Colombia.

##### Comment.

Coe (1951) named *Lineus stigmatus* from the shore of Biscayne Bay, Florida. He illustrates and describes in useful detail only the posterior portion, and this agrees strongly with the pattern of paired white markings seen in our specimen, which is not known from any other nemerteans in the region. We believe that it is reasonable to assign our specimens to Coe’s species; we base this on the distinctive color pattern and the strong regional distribution overlaps for many of the other regional nemertean species. Though Coe (1951) noted a potential similarity with *Lineus albocinctus* Verrill, 1900, we judge the color patterns to be very different. Coe (1951) also comments “…color pattern in this species has a superficial resemblance to that of some individuals of *Lineus geniculatus* (D. Chiaje) in which the white rings are interrupted in the mid dorsal line but in the latter species the rings continue laterally and ventrally”. Riser (1991) resurrected *Notospermus* (Huschke, 1830) and transferred to it *Lineus geniculatus* (*sensu* Delle Chiaje, 1828) of several authors. We agree that the pigment pattern resembles that of *Notospermus geniculatus* as well as that of a variety of similar worm images available via the internet and attributed to *Notospermus geniculatus* (JLN, pers obs). We anticipate that *Lineus stigmatus* is congeneric with *Notospermus geniculatus*, but this needs to be corroborated with internal anatomy and/or molecular data.

#### Micrura ignea

Taxon classificationAnimaliaHeteronemerteaLineidae

Schwartz & Norenburg, 2005

Fig. 3A–D

##### Description.

Two specimens up to about 100 mm long, 3 mm wide; dorsoventrally flattened; head “triangular”, pointed anteriorly and widening posteriorly; posterior end blunt with short and slender caudal cirrus. Orange grading posteriorly to yellowish. Deep cephalic furrows form lateral margins of head. Mouth slit-shaped, posterior to cephalic grooves. Eyespots lacking. Proboscis long, thick and pink; when everted it possesses ruffles.

##### Distribution.

Belize (Schwartz and Norenburg 2005); Caribbean coast of Panama (Collin et al. 2005); Santa Marta, Colombia.

### Hoplonemertea: Monostilifera

#### Amphiporus cruentatus

Taxon classificationAnimaliaHeteronemerteaMonostilifera

Verrill, 1879

Fig. 3E–G

##### Description.

Two specimens up to about 20 mm long, < 1 mm wide; dorsoventrally flattened, bluntly rounded at both ends. Pale yellow, with three thin, longitudinal blood vessels made bright red by corpuscles that can be observed flowing through the vessels. Cerebral organ furrows, vertical, not prominent, precerebral. About 6–10 conspicuous, blackish, precerebral ocelli, in single row along each. Rhynchopore subterminal at tip of head; rhynchocoel extends to about middle of body length; proboscis long and thick; armature approximately at center of proboscis; stylet slender (length: 30 µm), supported on cylindrical basis (33 × 8 µm); 2 pouches with 3 accessory stylets each. Mature females with dark or bright gray eggs visible through body wall.

##### Distribution.

Gulf of Mexico, New England (USA) and Washington (Norenburg 2009); California (Coe 1940); Santa Marta, Colombia.

#### Amphiporus cf. ochraceus

Taxon classificationAnimaliaHeteronemerteaMonostilifera

(Verrill, 1873)

Fig. 3H–J

##### Description.

Four specimens up to about 10 mm long, 0.5 mm wide; dorsoventrally flattened, bluntly rounded at both ends. Variable color, yellowish to milky gray, sometimes with light orange pigmentation in cephalic region corresponding to cerebral ganglia. Cerebral organ furrows vertical, precerebral. About 6-10 conspicuous, blackish, precerebral ocelli, arranged in single regular row, along each lateral margin of head. Rhynchopore subterminal; rhynchocoel about three quarters of body length; proboscis long; medially constricted basis same length as slender stylet (length: 29 µm); 2 pouches with 2 accessory stylets each.

##### Distribution.

Gulf of Mexico and New England (USA) (Norenburg 2009); Santa Marta, Colombia.

#### Amphiporus texanus

Taxon classificationAnimaliaHeteronemerteaMonostilifera

Coe, 1951

Fig. 3K–M

##### Description.

Four specimens up to about 15 mm long, 0.5 mm wide; dorsoventrally flattened, bluntly rounded at both ends; cephalic lobe narrower than foregut region. Dark brown body; with magnification, thick unpigmented margin (i.e., epidermis) appears white. Cerebral organ furrows vertical, precerebral. Row of about 6 ocelli present along each lateral margin of head; visible in squeeze preparation. Rhynchopore subterminal; proboscis large and thick, conspicuous papillae; central stylet slender (length: 42 µm), supported on wide cylindrical basis (34 × 10 µm) at middle of proboscis; two pouches with 2-4 accessory stylets each.

##### Distribution.

Gulf of Mexico and Southern Florida (Norenburg 2009); Curaçao (Schwartz and Norenburg 2005); Santa Marta, Colombia.

#### Nemertopsis bivittata

Taxon classificationAnimaliaHeteronemerteaMonostilifera

(Delle Chiaje, 1841)

Fig. 4A–C

##### Description.

One specimen up to about 20 mm long; 1 mm wide; rounded at both ends. Yellow milky base color, dorsally with 2 brown to reddish longitudinal lines joined at anterior and posterior ends. Cerebral organ furrows precerebral, difficult to see. Head almost undifferentiated from body. Cephalic grooves not visible. Two pre-cerebral eyes on each lateral margin of head. Rhynchopore subterminal; proboscis small, slender provided with papillae; short central stylet (length: 11 µm), supported on a massive base (27 × 7 µm). Two pouches containing three accessory stylet each.

##### Distribution.

USA East Coast – Florida (Thollesson and Norenburg 2003), South Carolina (Caplins et al. 2012); Atlantic Galician Island (Junoy and Herrera-Bachiller 2010); European waters, Portuguese and Spanish Exclusive Economic Zone, Red Sea (Gibson 2014b); Santa Marta, Colombia.

##### Comment.

Caplins et al. (2012) found support suggesting that the two color morphs of *Nemertopsis bivittata* commonly found sympatrically – one with dorsal stripes that meet anteriorly and the other with lines that do not meet – are genetically isolated. Support includes statistical difference in size of stylets and in DNA sequence differences for mitochondrial cytochrome-oxidase-1 gene – minimum and an a maximum pair-wise difference of 13.6% and 19.9% – (Caplins et al. 2012). Though awaiting explicit molecular data, Norenburg (2013) commented that *Nemertopsis bivittata* and *Nemertopsis gracilis* Coe, 1904, probably are synonyms and, therefore, following Sun and Dong (1998), the morph with lines meeting would be *Nemertopsis bullocki* Coe, 1940 and the one with stripes not meeting would be, by priority, *Nemertopsis bivittata* (Delle Chiaje, 1841). For now, we do not formally distinguish the two here and assign our specimen at *Nemertopsis bivittata*.

#### Ototyphlonemertes erneba

Taxon classificationAnimaliaHeteronemerteaMonostilifera

(Corrêa, 1950)

Fig. 4D

##### Description.

Up to about 12 mm long; < 1 mm wide; according to (Corrêa 1954), small meiofaunal worm dorsoventrally flattened, tapered at posterior end. Cream color. Cerebral organs and furrows present, precerebral. Eyes absent. Long sensory cirri along anterior and lateral margins of head. Pair statocysts; statolith an aggregation of 3 spherical granules. Rhynchocoel approximately half of body length; proboscis long, stout, with long papillae; anterior papillae (closest to proboscis insertion) each with rod-shaped inclusion; posterior proboscis opaque in transmitted light (white with incident light); stylet slender, > 2× length of pyriform basis; 2 pouches with 6 accessory stylets each.

##### Distribution.

Brazil (Corrêa 1950); Guajira, Colombia (Kirsteuer 1977).

#### Ototyphlonemertes lactea

Taxon classificationAnimaliaHeteronemerteaMonostilifera

(Corrêa, 1954)

Fig. 4E–G

##### Description.

Two specimens up to about 3.5 mm long; < 0.3 mm wide; truncated at both ends. Milky white color. Cerebral organs and associated furrows absent. Without ocelli. Two ovoid statocysts present, statolith formed by more than 10 spherical granules. Rhynchocoel short, about one-third of body length; proboscis very short; posterior proboscis vesicular (translucent) with transmitted light; armature at middle of proboscis; stylet slender, helically sculpted (length: 15 µm); basis thin, cylindrical (14 × 2 µm); 2 pouches with 3 accessory stylets each.

##### Distribution.

Brazil (Corrêa 1950); Guajira, Colombia (Kirsteuer 1977); Santa Marta, Colombia.

#### Zygonemertes fragariae

Taxon classificationAnimaliaHeteronemerteaMonostilifera

Corrêa, 1955

Fig. 4H–J

##### Description.

One specimen up to about 12 mm long, 1 mm wide; dorsoventrally flattened, bluntly rounded at both ends. Body vivid pinkish strawberry-red. Cerebral organ furrows shallow, precerebral. Pair irregular rows of 8-13 ocelli each on each side of head; row of about 10 ocelli between brain and each lateral margin of body and reaching post-cerebrally into foregut region. Rhynchopore subterminal; rhynchocoel to about middle of body length; proboscis long and thick with thin papillae; central stylet thin (length: 45 µm); basis smooth, cylindrical (54 × 14 µm); 2 pouches with 2 or 3 accessory stylets each. Epidermis with minute crescent-shaped intracellular spicules.

##### Distribution.

São Sebastião (Brazil) (Corrêa 1954); Santa Marta, Colombia.

#### Zygonemertes virescens

Taxon classificationAnimaliaHeteronemerteaMonostilifera

(Verrill, 1879)

Fig. 4K–M

##### Description.

Six specimens up to about 30 mm long, < 2 mm wide; dorsoventrally flattened, bluntly rounded at both ends. Variable color, from white to yellow or greenish. Cerebral organ furrows shallow, precerebral. Numerous pre-cerebral small ocelli arranged in two pair irregular rows of about 15–20 ocelli each; row of post-cerebral ocelli each side between brain and lateral margin of body, extending far posterior to brain along lateral nerve cord. Rhynchopore subterminal; rhynchocoel wide and almost full body length; proboscis long with small papillae; stylet slender (length: 60 µm) supported on massive and medially constricted basis (112 × 28 µm); two pouches, each bearing 3 accessory stylets. Epidermis with minute crescent-sphaped intracellular spicules.

##### Distribution.

Gulf of Mexico and New England (Norenburg 2009); California and Oregon (Corrêa 1964); Southern Florida and Virgin Islands (Corrêa 1961); Gulf of Maine (Gibson 2014c); North Atlantic (Azores) (Strand 2002); Santa Marta, Colombia.

### 4-eyed monostiliferan sp. 1

Fig. 5A–C

**Description.** Two specimens about 12 mm long, 1 mm wide; dorsoventrally flattened. White to yellow-brownish color. Cerebral organ furrows shallow, precerebral. Four precerebral ocelli set as corners of a wide rectangle. Rhynchocoel voluminous, extending almost full body length. Rhynchopore subterminal. Proboscis stout; armature far posterior; Stylet (length 45 µm) supported on massive medially constricted basis (110 × 50 µm); two pouches with 2 accessory stylets each.

**Distribution.** Santa Marta, Colombia.

### 4-eyed monostiliferan sp. 2

Fig. 5D–E

**Description.** One specimen about 10 mm long, < 1 mm wide; dorsoventrally flattened; head arrow-shaped, tail tapered. Milky white color. Brain appears as a pink orange spot in head. Cerebral organ furrows shallow, precerebral. Postcerebral groove inconspicuous, forms dorsal “V”. Two pair ocelli, anterior and posterior separated by cerebral organ furrow. Rhynchopore subterminal. Proboscis stout, densely papillated.

**Distribution.** Santa Marta, Colombia.

### 4-eyed monostiliferan sp. 3

Fig. 5F–H

**Description.** One specimen about 6 mm long, < 1 mm wide; dorsoventrally flattened; head arrow-shaped; tail tapered. Cream to greenish color. Brain region pink. Cerebral organ furrows precerebral, deep. Postcerebral groove forms a “V” dorsally. Two pair ocelli, set as square, anterior and posterior separated by cerebral organ furrows. Rhynchopore subterminal; rhynchocoel extends three fourths of body length. Stylet (length: 70 µm) supported on massive pear-shaped basis (130 × 60 µm).

**Distribution.** Santa Marta, Colombia.

### Cratenemertidae sp.

Fig. 5I–K

**Description.** Two specimens up to about 22 mm long, 2 mm wide; tapered at both ends. Uniform bright red color. Conspicuous mid-dorsal cephalic crest. Cerebral organ furrows precerebral, inconspicuous, with few faint ridges orthoganol to furrow axis. About 26 ocelli scattered in four elongate, irregular groups, anterior and posterior separated by cerebral organ furrows. Rhynchopore subterminal. Proboscis long and stout, with dense, large papillae; stylet (length: 120 µm) on short, wide and rounded basis (50 × 48 µm); two pouches containing three accessory stylets each. Worms capable of swimming with strong undulating movements.

**Distribution.** Santa Marta, Colombia.

### Hoplonemertea: Polystilifera

#### Punnettia cf. natans

Taxon classificationAnimaliaHoplonemerteaPolystilifera

(Kirsteuer, 1973)

Fig. 5L–M

##### Description.

One specimen about 17 mm long, < 1 mm wide; dorsoventrally flattened, tapered at both ends. Gray to brown color, darker on head and along mid-dorsal line of body; ventral surface milky gray. Head narrow with respect to body. Cerebral organ furrows wide, postcerebral, subdivided by about 5 longitudinal epithelial ridges (secondary furrows) orthoganol to furrow axis. Numerous large ocelli, precerebral, arranged in four irregular longitudinal rows, outer rows possibly divided into anterior and posterior clusters. Armature normally several stylets supported on a single basis, but not documented by us. Individuals capable of swimming by undulatory movements, leaving mucus behind it.

##### Distribution.

Bahía Mochima (Venezuela) (Kirsteuer 1973); Santa Marta, Colombia.

##### Comment.

Two named polystiliferan species are known from the region. *Polyschista curacaoensis* Stiasny-Wijnhoff, 1925 is known only from three pieces – two heads and a tail – already preserved and strongly contracted when first examined by Stiasny-Wijnhoff; therefore, of dubious value for anatomical study. The heads are described as having a “a well defined brown longitudinal marking on their back” while “the margins are a milky, transparent white and two to three times as broad as the thick [brown] middle part”. The tail is described as being transparent and white. This does not fit well the present species. *Curranemertes natans* Kirsteuer, 1973 is known only from Venezuela and described as “orange to brownish” dorsally, with the thicker median region of the head being a “darker brownish shade”. Though Kirsteuer (1973) concludes that the two species differ in internal anatomy, he cites only character states that he presumes “probably” differ in *Polyschista*. We are not sanguine that our specimen is either species, though it resembles specimens previously collected by JLN off Belize and Bocas del Toro, Panama and identified as *Curranemertes* cf. *natans*.

Härlin (1998), with support from a morphological phylogenetic analysis re-assigned *Curranemertes natans* to the phylo-clade *Punnettia*. Härlin and Härlin (2000) found *Punnettia* to be paraphyletic but without placing the type species, *Punnettia hubrechti*. They concluded by discarding *Punnettia*, in a Phylocode act, with the argument that it is “a messy name” and assigning its species to two phylo-clades but without placing *Punnettia hubrechti*. Thus, with respect to a Linnean classification, we cannot know which *Punnettia* species would remain so, because they form a clade with *Punnettia hubrechti*. Hence, it seems more appropriate at this time to retain *Curranemertes natans*, pending resolution of *Punnettia* phylogeny.

## Comments

Direct observations of the nemerteans “in vivo” facilitates collection of information about nemertean species that is more reliable than possible with preserved specimens, and permits photographic records useful for their identification (Norenburg 2009). Taxonomy of four-eyed monostiliferans is difficult because many of the species described were, historically, mis-allocated and many of the descriptions lack useful characterization of external features, and even characterization of internal anatomy often is of dubious quality and misleading (JLN pers obs). Recognition and allocation of species that lack highly distinctive diagnostic features, such as those in this study, is especially difficult. While internal anatomy could point to recently formulated generic diagnoses, the work involved is better suited to individual monographic studies.

Although, the purpose of this research was taxonomical, it is worth mentioning that the most frequent species was *Dushia atra*, representing 30% of the total of collected specimens. It was followed by *Baseodiscus delineatus* and *Zygonemertes virescens* at 12% each. The major diversity, in terms of number of species, was observed at Inca-Inca, with 7 species. However, the sampling effort was not the same in all collection sites, so it is not possible to make a reliable comparison of the biodiversity among the stations. Most studies of nemerteans in the Caribbean, and elsewhere, have focused on taxonomy (Coe 1901; Corrêa 1961, 1963; Kirsteuer 1973, 1974, 1977; Schwartz and Norenburg 2005; Collin et al. 2005), and there are no other records of relative abundances or dominance of species.

Frequently, aggregations of 3–5 con-specific specimens of *Amphiporus cruentatus*, *Amphiporus texanus*, *Dushia atra* and *Zygonemertes virescens*, were found under rocks or in rock crevices. This behavior has been observed before in several species of nemerteans and in some cases may be related with reproduction events (Thiel and Junoy 2006) but often it seems the worms are gregarious, though that may be a by-product of worms focusing on particularly suitable microhabitats (JLN, pers obs).

This study represents the first taxonomic work focused on nemerteans of Colombia, and specifically the Caribbean coast. Except for *Ototyphlonemertes lactea*, all species are new records for Colombia. Among the 36 species reported from the Caribbean region (Corrêa 1961, 1963; Kirsteuer 1974, 1977; Schwartz and Norenburg 2005) at least 12 are present in the rocky shores of two beaches in the small region of Santa Marta. This suggests that more intensive investigation, across more habitats, will yield significantly greater nemertean diversity. This study also begins to open up study of nemerteans in Colombia to different fields of biology.

## Supplementary Material

XML Treatment for Tubulanus rhabdotus

XML Treatment for Baseodiscus delineatus

XML Treatment for Dushia atra

XML Treatment for Lineus stigmatus

XML Treatment for Micrura ignea

XML Treatment for Amphiporus cruentatus

XML Treatment for Amphiporus cf. ochraceus

XML Treatment for Amphiporus texanus

XML Treatment for Nemertopsis bivittata

XML Treatment for Ototyphlonemertes erneba

XML Treatment for Ototyphlonemertes lactea

XML Treatment for Zygonemertes fragariae

XML Treatment for Zygonemertes virescens

XML Treatment for Punnettia cf. natans
